# Long-term ecosystem development and retrogression drive microbial specialization for complex organic matter degradation

**DOI:** 10.1093/ismeco/ycag157

**Published:** 2026-06-07

**Authors:** Flúvio Modolon, Eric Capo, David A Wardle

**Affiliations:** Department of Ecology, Environment and Geoscience, Umeå University, Umeå, Västerbotten 90187, Sweden; Department of Ecology, Environment and Geoscience, Umeå University, Umeå, Västerbotten 90187, Sweden; Department of Ecology, Environment and Geoscience, Umeå University, Umeå, Västerbotten 90187, Sweden

**Keywords:** boreal forest, ecosystem retrogression, *Actinomycetota*, metagenome-assembled genomes (MAGs), decomposition, Chronosequence, soil microbial specialization, organic matter breakdown, complex carbohydrate degradation

## Abstract

Long-term ecosystem development includes a build-up phase followed by a decline (retrogressive) phase characterized by reduced plant productivity and belowground process rates due to reduced nutrient availability. In boreal forests, retrogression is accompanied by soil organic matter (SOM) accumulation, especially in the prolonged absence of fire. However, the role of bacterial communities in SOM dynamics during ecosystem retrogression has been little explored. Using a 5000-year post-fire boreal forest chronosequence, we investigated how long-term succession and retrogression shapes soil bacterial community structure and functional specialization. While the *Actinomycetota* phylum dominated communities across all chronosequence stages, a significant family-level shift within this phylum occurred in the later (retrogressive) phase, characterized by a transition from *Mycobacteriaceae* to *Streptosporangiaceae*. The recovery of metagenome-assembled genomes (MAGs) revealed distinct life-history trade-offs between these families. *Streptosporangiaceae* MAGs were significantly enriched in genes for degrading phenolics, cellulose, and lignin, and exhibited potential for chitin, lipid and peptide degradation. This positions them as potential decomposers of the primary constituents of stored soil carbon, including plant-derived complex carbohydrates and fungal necromass, during retrogression when fungal activity declines. In contrast, *Mycobacteriaceae* MAGs are likely to prioritize inorganic phosphate (P*_i_*) uptake-by *pstS* gene enrichment, reflecting adaptation to P availability changes during ecosystem development. Collectively, our results demonstrate that long-term ecosystem retrogression drives shifts in the bacterial communities and functions within the *Actinomycetota.* These shifts may indicate possible divergent strategies, i.e. recalcitrant carbon turnover versus nutrient scavenging, which could explain shifts in the microbial community as the ecosystem transitions toward retrogressive, nutrient-limited states.

Long-term ecological succession and ecosystem development generally progresses from an initial phase of biomass accumulation to a retrogressive phase after thousands of years, characterized by declining plant productivity and belowground process rates due to reduced nutrient availability [1]. Studies across contrasting long-term chronosequences worldwide reveal that these shifts during retrogression are accompanied by changes in soil microbial community composition and physiology [2–5]. In boreal forests, retrogression—which occurs in the absence of fire over thousands of years—leads to substantial soil organic matter (SOM) accumulation, partly due to diminished decomposer activity and the production and buildup of recalcitrant fungal necromass [6, 7]. Some recent studies have explored the role of the fungal community in driving belowground processes that lead to SOM buildup during retrogression [6–8]. However, although gram-positive bacteria—known to preferentially exploit complex C sources in organic soils—increase during retrogression in boreal forests [9], the role of bacterial players and their functional strategies in SOM dynamics during retrogression remains largely unexplored. Here, using a well characterized boreal forest post-fire chronosequence that spans ~5000 years, we provide the first genomic-centric view of taxonomic and functional shifts of the bacterial community during ecosystem retrogression and interpret these patterns in terms of bacterial adaptations for nutrient acquisition and the decomposition of recalcitrant SOM.

We investigated a well-characterized fire-driven boreal forest chronosequence on forested islands in Lakes Hornavan and Uddjaure, Sweden [10–12] that provides a natural experiment to test how long-term retrogression shapes microbial community structure and functional shifts. Island size determines fire frequency: larger islands are more likely to intercept lightning strikes and therefore burn more frequently, whereas smaller islands can remain undisturbed for several millennia, generating a strong natural gradient in time since fire without major geological or climatic confounders. With increasing time since fire, retrogression proceeds due to progressive nutrient limitation, leading to declining litter quality and increasingly recalcitrant fungal necromass and ultimately greatly increased belowground C sequestration [6, 7, 12]. Soil organic layers deepen progressively, exceeding 1 m on the smallest islands, and belowground C accumulates linearly at the rate of 0.5 kg/m^2^ for every century without fire [11]. Consistent with earlier work in this system, we focused on 30 lake islands (10 in each of three size classes), hereafter referred to as “large” islands (>1.0 ha; time since fire = 585 years), “medium” islands (0.1–1.0 ha; 2180 years) and small islands (<0.1 ha; 3250 years) [6, 11]. Further details on the study system are given in Table S1.

We applied shotgun metagenomic sequencing to perform taxonomic compositional profiling and reconstruction of metagenome-assembled genomes (MAGs) (see Supplemental Information for details). Across all island size classes, the *Actinomycetota* phylum was dominant, with relative abundances ranging from 51.6% (SD ±14.6) to 63.0% (±14.3), and no significant differences among island sizes (Kruskal-Wallis, p > 0.05). At the family level, *Mycobacteriaceae* and *Streptosporangiaceae* were the dominant actinomycetes. Small islands had significantly more *Streptosporangiaceae* relative to medium (log₂ fold difference = 1.91) and large islands (2.62) and significantly less *Mycobacteriaceae* than did medium (−1.19; Fig. 1a–d). Overall taxonomic richness was significantly greater on small than on medium, or large islands (Fig. 1e), and overall community structure of small islands also differed significantly from that of both medium and large islands (Fig. 1f, Table S2).

**Figure 1:**
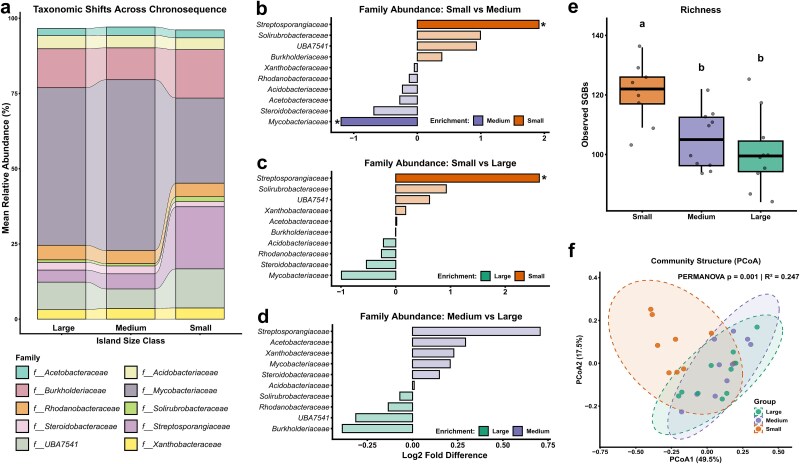
Microbial community structure and successional dynamics across a 5000-year post fire boreal forest chronosequence. (a) Mean relative abundance of top 10 most abundant microbial families across the three island size classes. (b–d) Differential abundance of the top 10 most abundant microbial families across pairs of island size classes, calculated using DESeq2. Bars represent the log₂ fold difference in abundance between island size classes, with colors indicating enrichment in a specific group on one island size class relative to the other: (b) small (orange) vs. medium (purple); (c) small (orange) vs. large (teal); and (d) medium (purple) vs. large (teal). Asterisks (*) indicate significant differences between pairs of island size classes (Wald test, Benjamini-Hochberg adjusted *P* < .05). (e) taxonomic richness across island size classes. Significant differences (*P* < .05) are indicated by distinct letters (a, b) determined by a Kruskal-Wallis test followed by a Dunn’s post-hoc test. f, principal coordinates analysis (PCoA) based on Bray–Curtis dissimilarity, visualizing variation in community structure at the species level. Points represent individual islands, and ellipses indicate 95% confidence intervals for of the three island size groups. PERMANOVA results confirm statistically significant differences in community composition between groups.

We selected 29 medium- to high-quality *Actinomycetota* MAGs for downstream analysis, including 17 *Mycobacteriaceae* and 12 *Streptosporangiaceae*, given that these groups were enriched on medium-large, and on small, islands, respectively (Supplementary Data S1). Functional inference targeted KEGG orthologs (KOs) encompassing genes related to nutrient acquisition (C fixation, N and P cycling) and OM decomposition. Gene enrichment analyses (log_2_ fold difference in KO count between MAG families) identified significant differences (Wald’s test) in KO abundances between these two families (Fig. 2a and b).

**Figure 2:**
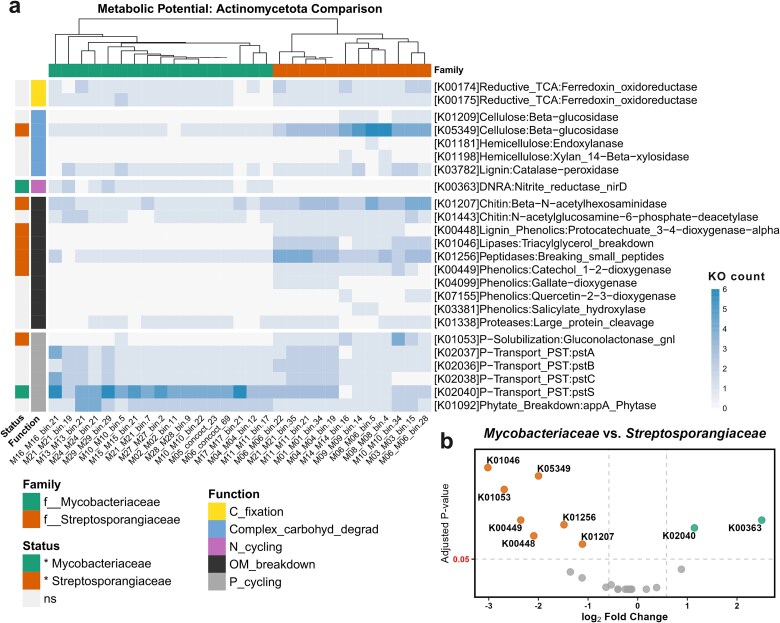
Functional inference for metabolic specialization of *Mycobacteriaceae* and *Streptosporangiaceae* MAGs. The heatmap, (a) represent the distribution and copy number of specific KOs across 29 high-quality *Actinomycetota* metagenome-assembled genomes (MAGs). Color intensity (white to blue) indicates the copy number of each KO within the respective MAG. Columns represent individual MAGs assigned to the families *Mycobacteriaceae* (dark-teal bar) and *Streptosporangiaceae* (orange bar). Rows represent functional genes categorized by function (“function” column on left of figure), including “carbon fixation” (yellow), “complex carbohydrate degradation” (light blue), “nitrogen cycling” (purple), “organic matter breakdown” (black), and “phosphorus cycling” (grey). Dark-teal “status” bar represents the KOs statistically significantly enriched for *Mycobacteriaceae* while orange “status” bar represents the significant KOs for *Streptosporangiaceae* (Wald test, Benjamini-Hochberg, adjusted *P* < .05). The volcano plot, (b), shows statistical support for the differential enrichment of KOs between the two families. Each dot represents a specific KO, with the x-axis denoting the log_2_ fold difference in the KO copy number between *Mycobacteriaceae* and *Streptosporangiaceae* and the y-axis shows corresponding statistical significance of differential KO enrichment between the two families (Wald test, Benjamini-Hochberg, adjusted *P* < .05). KOs significantly enriched for *Streptosporangiaceae* and *Mycobacteriaceae* are shown in orange and green dots, respectively. KOs not meeting both the adjusted *P*-value (< .05) and effect size (log_2_ fold difference > 0.58) thresholds are shown in grey.

MAGs assigned to *Streptosporangiaceae* were significantly enriched in KOs involved in the degradation of complex and recalcitrant SOM (Fig. 2a). These included traits associated with the breakdown of phenolic compounds and complex carbohydrates such as cellulose and lignin. Notably, several orthologs involved in phenolic compound degradation were exclusively detected in *Streptosporangiaceae*. This pattern is consistent with ecosystem-scale shifts in substrate chemistry along the chronosequence. For instance, as time since fire increases, soils accumulate higher concentrations of polyphenolics (including tannins and derivates) [10]. These compounds, largely derived from litter from late-successional, resource-conservative plant species, bind nitrogen into protein–phenolic complexes and contribute to humus accumulation by impairing decomposition [12]. The enrichment of KOs for phenolic degradation enzymes in *Streptosporangiaceae* therefore suggests adaptation to the high SOM recalcitrance characteristic of retrogressive ecosystems. Additionally, *Streptosporangiaceae* MAGs were enriched in orthologs related to cellulose and lignin breakdown, consistent with the increased relative production of plant-derived structural compounds observed in smaller islands [13].

*Streptosporangiaceae* also showed enrichment in orthologs for chitinases and peptidases which could confer an advantage on the small islands. Given that the majority of the soil carbon on small islands is derived from root-associated fungal necromass, especially from melanized ericoid mycorrhizal fungal hyphae [6], enrichment in chitinolytic and proteolytic traits in *Streptosporangiaceae* may help them utilize such substrates. The genomic evidence suggests that *Streptosporangiaceae* could potentially contribute to the transformation of C from recalcitrant fungal necromass into more labile prokaryotic biomass, which is likely to turn over more rapidly [14, 15]. However, the high C storage observed in these soils suggests that even with the activity of *Streptosporangiaceae*, overall turnover rates remain low.

*Mycobacteriaceae* MAGs, in contrast, were significantly enriched in KOs related to phosphorus (P) uptake, especially for the high-affinity phosphate-binding protein (*pstS*) which is part of the P*_i_* import system (*pstSABC*). Across the island chronosequence, ecosystem retrogression is characterized by declining availability of overall P pools as it gets incorporated into OM and biomass or lost from the system [12, 16]. The enrichment of *pstS* in *Mycobacteriaceae* likely explains their greater abundance in earlier successional stages, mirroring, to some extent, the prevalence on large islands of cord-forming ectomycorrhizal fungi that are known to be effective at P uptake [7]. It is important to note, however, that the P bound in SOM requires additional enzymatic steps to be converted into P*_i_*, and that these steps may be carried out by other microbes.

Collectively, our findings are indicative of functional differentiation among actinomycetal taxa in nutrient assimilation and C cycling across a boreal forest chronosequence and during retrogression. We also provide a novel bacterial perspective that complements the predominantly fungal-centric framework of ecosystem retrogression in boreal forests, by identifying bacterial taxa that are linked to the breakdown of complex SOM forms and P*_i_* uptake. These results suggest that specialized actinomycetes may emerge as critical functional drivers during long-term retrogression as fungal decomposer activity declines. More broadly, boreal forests store more C than any other forest biome—most of it belowground [17] - and shifts in fire regimes driven by climate change will impact their C sink potential [18]. In this context our results highlight the importance of understanding the role of these bacterial drivers in shaping the long-term stability of this C sink in a changing global environment.

## Supplementary Material

Supplementary_material_ycag157

## Data Availability

Raw sequence data generated for this are publicly available in the National Centre for Biotechnology Information (NCBI) database under the BioProject PRJNA1474501. The metagenome-assembled genomes (MAGs) reconstructed and analyzed in this study are available in the Figshare repository at https://doi.org/10.6084/m9.figshare.31812181. The codes used for analyses and figure generation, as well as the input data and metadata analyzed, are available in the GitHub repository, https://github.com/modolon/Meta_boreal_retrogression.
