# Supplementary material for: Long-term ecosystem development and retrogression drive microbial specialization for complex organic matter degradation
Source: ISME Commun. 2026 Jun 7;6(1):ycag157. doi: 10.1093/ismeco/ycag157 (PMC13333084; doi:10.1093/ismeco/ycag157)
Supplement: Supplementary_material_ycag157 [file supplementary_material_ycag157.zip › Supplemental_Modolon-Capo-Wardle_Reviewed-cleaned_May15.docx]

**SUPPLEMENTAL DATA**

**Table S1.** Measurements of selected ecosystem properties (mean values ± standard errors) measured at the whole island scale for the three island size classes (N = 10 islands per class). Within each row, numbers followed by the same letter are not statistically significant at α = 0.05 (Tukey’s test following one-way ANOVA). Data from Gundale et al. (2011), Lagerström et al. (2009), Wardle and Zackrisson (2005) and Wardle et al. (1997, 2003, 2004, 2012).

| Ecosystem property | Island size | | |
| --- | --- | --- | --- |
|  | Small | Medium | Large |
| Time since last fire (years) | 3250 ± 439 a | 2180 ± 385 b | 585 ± 233 c |
| Humus carbon storage (g/m^2^) | 27.2 ± 2.5 a | 16.2 ± 2.5 b | 6.4 ± 1.1 c |
| Net primary productivity (g/m^2^/yr) | 159 ± 18 b | 247 ± 12 a | 256 ± 14 a |
| Standing plant biomass (g/m^2^) | 3470 ± 470 b | 8340 ± 877 a | 9349 ± 485 a |
| Vascular plant species richness* | 10.6 ± 0.6 a | 8.6 ± 0.4 b | 6.6 ± 0.6 c |
| Humus C to N ratio | 32.9 ± 0.79 b | 36.0 ± 1.17 ab | 40.4 ± 1.18 a |
| Humus C to P ratio | 759 ± 30 a | 687 ± 36 ab | 623 ± 20 b |
| Humus N to P ratio | 23.3 ± 1.1 a | 19.1 ± 0.9 b | 15.4 ± 0.5 c |
| MIN/(MIN+DON) | 0.39 ± 0.03 b | 0.53 ± 0.05 a | 0.49 ± 0.04 a |
| Mineral P (µgP/g) | 24.4 ± 2.3 b | 37.7 ± 4.3 a | 43.6 ± 4.9 a |
| Membrane-extractable P (mmol/kg) | 4.9 ± 0.3 b | 6.5 ± 0.4 a | 5.9 ± 0.7 ab |

^*^Number of species in a 10 m radius circular plot

**Table S2.** Results of pairwise Permutational Multivariate Analysis of Variance (PERMANOVA) based on Bray-Curtis dissimilarities comparing microbial community composition between pairs of island size categories.

| Pairs | Df | Sums of Sqs | F Model | R^2^ | p-value | p-adjusted |
| --- | --- | --- | --- | --- | --- | --- |
| Medium vs Small | 1 | 0.490 | 6.543 | 0.278 | 0.003 | 0.009 |
| Medium vs Large | 1 | 0.032 | 0.463 | 0.025 | 0.789 | 1.000 |
| Small vs Large | 1 | 0.464 | 5.530 | 0.245 | 0.002 | 0.006 |

**SUPPLEMENTAL METHODS**

**Sampling**

Soil samples were collected from each of the 30 islands between July 31 and August 12, 2025. For each island 25 individual soil (humus) cores, each 2.5 cm diameter and 10 cm deep, were collected within a defined 20 m x 20 m area in the vicinity of previous work on these islands (Wardle et al. 2012, Clemmensen et al. 2013, 2015) and bulked within each island as individual islands served as the units of replication. During the field campaign, samples were maintained at 4°C up to 5 days. Upon arrival at the laboratory, the soils were transferred to -20°C for storage until DNA extraction. Prior to processing, samples were sieved to 4 mm and homogenized to ensure a uniform composite mixture.

**Sample Processing and Sequencing**

For each sample total genomic DNA was extracted from 0.25g of soil samples using the DNeasy PowerSoil Pro Kit (Qiagen), following the manufacturer’s protocol, implementing 10 min vortex and 50 μL for the final elution. For each sample, library preparation was initiated with 50 ng of DNA using the SMARTer ThruPLEX DNA-seq Kit (Takara Bio). Fragmentation was conducted using a Covaris E220 system to achieve a target insert size of 350–400 bp. Libraries were uniquely indexed using Unique Dual Index Sets A–D (Takara Bio) to prevent index hopping and ensure high-multiplexing accuracy, following the manufacturer’s instructions. The resulting libraries were sequenced on the Illumina NovaSeq X Plus platform using a 25B flow cell at NGI SciLifeLab (Uppsala, Sweden).Sequencing was performed with XLEAP-SBS chemistry to generate paired-end 150 bp reads. This configuration was selected to provide the high throughput and base-call accuracy required for deep metagenomic profiling and the reconstruction of high-quality Metagenome-Assembled Genomes (MAGs).

**Bioinformatics**

Raw metagenomic reads were processed for quality control and adapter removal using fastp v0.23.4 (Chen et al., 2018). Read quality was assessed before and after trimming using FastQC v0.12.1 (Andrews, 2010), with final reports consolidated via MultiQC v1.22.3 (Ewels et al., 2016). Clean reads from each soil sample were individually assembled into contigs using the *de novo* assembler MEGAhit v1.2.9 (Li et al., 2015). Clean reads were mapped back to their respective contig assemblies using Bowtie2 v2.5.4 (Langmead & Salzberg, 2012), and SAMtools v1.22 (Danecek et al., 2021). Contig coverage was calculated using the tool jgi_summarize_contig_depths from MetaBAT2 v2.17 (Kang et al., 2019).

To provide a comprehensive view of the microbial community, taxonomic profiling of the whole metagenome was performed with MetaPhlAn v4.1.1 (Blanco-Míguez et al., 2023), which generated a taxonomy table with relative abundance data. Metagenome-assembled genomes (MAGs) were reconstructed using a consensus-based approach. Initial binning was performed on each sample using three independent algorithms: MetaBAT2 v2.17, CONCOCT v1.1.0 (Alneberg et al., 2014), and MaxBin2 v2.2.7 (Wu et al., 2016). The resulting bin sets were integrated and refined into a non-redundant set of high-quality bins using DAS_Tool v1.1.7 (Sieber et al., 2018). The quality of the refined MAGs, including completeness and contamination, was assessed using the machine-learning-based tool CheckM2 v1.1.0 (Chklovski et al., 2023). Taxonomic assignment for all recovered MAGs was performed using GTDB-Tk v2.6.1 (Chaumeil et al., 2022) against the GTDB release 10-RS226 (Parks et al., 2022). For functional characterization, open reading frames (ORFs) were predicted using Prodigal v2.6.3 (Hyatt et al., 2010), and functional annotation was conducted using KofamScan v1.3.0 (Aramaki et al., 2020) based on the Kyoto Encyclopedia of Genes and Genomes (KEGG) database. All taxonomic outputs were standardized to the GTDB nomenclature to ensure consistency across analyses.

**Statistical Analysis**

*Differential Abundance Analysis*

To identify differences in relative abundances at the family level across each of the three possible pairs of island size classes, (i.e., large vs medium, large vs small, medium vs small), we used the Wald test implemented in the DESeq2 package (Love et al., 2014) in R v4.4.1. The input for this analysis was a count matrix with rows representing microbial families and columns representing individual islands, and it was generated by scaling MetaPhlAn4-derived relative abundances by a factor of 10,000 and rounding to the nearest integer. The matrix was then filtered to retain the 10 most abundant families, and for each family the difference in abundance between the two island size classes for each pair was evaluated by calculating the log_2_ fold differences in counts between the two size classes. Statistical significance of this difference was determined using Benjamini-Hochberg adjusted p-values (p_adj_ < 0.05). Visualizations were generated using ggplot2 (Wickham, 2016).

*Comparative Metabolic Potential of MAGs*

To investigate functional shifts between key actinomycetal lineages (*Mycobacteriaceae* vs. *Streptosporangiaceae*), we analyzed the distribution of KEGG Orthologs (KOs) across recovered MAGs. Count data for metabolic functions were filtered to include only MAGs of medium-to-high quality, following the criteria defined by Bowers et al. (2017): high quality, >90% completeness and < 5% contamination; medium quality, ≥50% completeness and less < 10% contamination; low quality, <50% completeness and <10% contamination. Differential enrichment of functional genes was assessed using DESeq2, employing the poscounts size factor estimation to account for the sparse nature of the functional matrix. Functional genes (KOs) were considered significantly enriched in one lineage relative to the other if they exhibited a log_2_ fold change > 0.58 in KO counts (representing at least a 1.5-fold difference in abundance) and a Benjamini-Hochberg adjusted p-value (p_adj_ < 0.05). Heatmaps showing the metabolic potential and significance status were generated using pheatmap R package (Kolde, 2019), with hierarchical clustering of columns (MAGs) based on Euclidean distance.

Preliminary differential enrichment of functional genes was also performed on the full unbinned assembly (MEGAhit outputs) to assess the influence of island size on gene distribution. However, this approach yielded lower biological resolution and higher background noise than did the MAG-centric analysis, which provided more robust links between specific taxonomic shifts and metabolic strategies. For this reason, our study focused on the results from the latter analysis.

*Diversity and Community Structure Analysis*

Taxonomic richness comparisons were conducted using the vegan package (Oksanen et al., 2022) at the species-level genome bin (SGB) level (taxonomic unit used in MetaPhlan4 output). Overall differences in richness among size classes were evaluated with a Kruskal-Wallis test, followed by Dunn’s test with Bonferroni correction for pairwise comparisons. Differences in overall community structure among island size classes were assessed using Bray-Curtis dissimilarity on proportion data. Community separation was visualized through Principal Coordinates Analysis (PCoA). Statistical significance among island size classes was tested using Permutational Multivariate Analysis of Variance (PERMANOVA) via the adonis2 function (999 permutations). Additionally, the homogeneity of multivariate dispersions was verified using the betadisper and permutest functions to ensure that observed differences were due to community shifts rather than variance heterogeneity.

**Supplemental references**

Alneberg J, et al. Binning metagenomic contigs by coverage and composition. *Nat Methods* 2014;11:1144–1146.

Andrews S, et al. FastQC: a quality control tool for high throughput sequence data. 2010.

Aramaki T, et al. KofamKOALA: KEGG ortholog assignment based on profile HMM and automated thresholding. *Bioinformatics* 2020;36:2251–2252.

Blanco-Míguez A, et al. Extending and improving metagenomic taxonomic profiling with MetaPhlAn 4. *Nat Biotechnol* 2023;41:1633–1644.

Bowers, RM., et al. (2017). Minimum information about a single amplified genome (MISAG) and a metagenome-assembled genome (MIMAG) of bacteria and archaea. *Nat Biotechnol* 2017; 35: 725-731.

Chaumeil PA, et al. GTDB-Tk v2: a standardized toolkit for assigning software-independent taxonomy to genomes. *Bioinformatics* 2022;38:5315–5316.

Chen S, et al. fastp: an ultra-fast all-in-one FASTQ pre-processor. *Bioinformatics* 2018;34:i884–i890.

Chklovski A, et al. CheckM2: a rapid, scalable and accurate tool for assessing microbial genome quality using machine learning. *Nat Methods* 2023;20:1203–1212.

Danecek P, et al. Twelve years of SAMtools and BCFtools. *GigaScience* 2021;10:giab008.

Ewels P, et al. MultiQC: summarize analysis results for multiple tools and samples in a single report. *Bioinformatics* 2016;32:3047–3048.

Graves S, et al. multcompView: Visualizations of Paired Comparisons. *R package version 0.1-8* 2019.

Gundale MJ, et al. Resource heterogeneity does not explain the diversity–productivity relationship across a boreal island fertility gradient. *Ecography* 2011;34:887–896.

Hyatt D, et al. Prodigal: prokaryotic gene recognition and translation initiation site identification. *BMC Bioinformatics* 2010;11:119.

Kang DD, et al. MetaBAT 2: an adaptive binning algorithm for robust and efficient genome reconstruction from metagenome assemblies. *PeerJ* 2019;7:e7359.

Kolde R, et al. pheatmap: Pretty Heatmaps. *R package version 1.0.12* 2019.

Lagerström A, et al. Soil phosphorus and microbial response to a long-term wildfire chronosequence in northern Sweden. *Biogeochemistry* 2009;95:199–213.

Langmead B, et al. Fast gapped-read alignment with Bowtie 2. *Nat Methods* 2012;9:357–359.

Li D, et al. MEGAhit: an ultra-fast single-node solution for large and complex metagenomics assembly via succinct de Bruijn graph. *Bioinformatics* 2015;31:1674–1676.

Love MI, et al. Moderated estimation of fold change and dispersion for RNA-seq data with DESeq2. *Genome Biol* 2014;15:550.

Oksanen J, et al. vegan: Community Ecology Package. *R package version 2.6-4* 2022.

Parks DH, et al. GTDB: an ongoing community effort with molecular lineages and a unified taxonomy. *Nucleic Acids Res* 2022;50:D785–D794.

Sieber CM, et al. Recovery of genomes from metagenomes via a optimized integration of binning algorithms. *Nat Microbiol* 2018;3:836–843.

Wardle DA, et al. The influence of island area on ecosystem properties. *Science* 1997;277:1296–1299.

Wardle DA, et al. Long-term effects of wildfire on ecosystem properties across an island area gradient. *Science* 2003;300:972–975.

Wardle DA, et al. Ecosystem properties and forest decline in contrasting long-term chronosequences. *Science* 2004;305:509–513.

Wardle DA, et al. Effects of species and functional group loss on island ecosystem properties. *Nature* 2005;435:806–810.

Wardle DA, et al. Linking vegetation change, carbon sequestration and biodiversity: insights from island ecosystems in a long‐term natural experiment. *J Ecol* 2012;100:16–30.

Wickham H, et al. ggplot2: Elegant Graphics for Data Analysis. *Springer-Verlag* 2016.

Wu YW, et al. MaxBin 2.0: an automated binning algorithm to recover genomes from multiple metagenomic datasets. *Bioinformatics* 2016;32:605–607.
